# Isolation and genomic characterization of a *Dehalococcoides* strain suggests genomic rearrangement during culture

**DOI:** 10.1038/s41598-017-02381-0

**Published:** 2017-05-22

**Authors:** Masafumi Yohda, Kentaro Ikegami, Yuto Aita, Mizuki Kitajima, Ayane Takechi, Megumi Iwamoto, Tomomi Fukuda, Noriyoshi Tamura, Junji Shibasaki, Seiji Koike, Daisuke Komatsu, Sakari Miyagi, Minoru Nishimura, Yoshihito Uchino, Akino Shiroma, Makiko Shimoji, Hinako Tamotsu, Noriko Ashimine, Misuzu Shinzato, Shun Ohki, Kazuma Nakano, Kuniko Teruya, Kazuhito Satou, Takashi Hirano, Osami Yagi

**Affiliations:** 1 Department of Biotechnology and Life Science, Tokyo University of Agriculture and Technology, Koganei, Tokyo 184-8588 Japan; 2 PaGE Science, Koganei, Tokyo 184-8588 Japan; 3 ADEKA Co., Ltd., Arakawa, Tokyo 116-8554 Japan; 4 In Situ Solutions, Co., Ltd., Chiyoda, Tokyo 101-0044 Japan; 5 Biological Resource Center, National Institute of Technology and Evaluation, Shibuya, Tokyo 151-0066 Japan; 6 Okinawa Institute of Advanced Sciences, Uruma, 904-2234 Okinawa Japan; 7 Department of Applied Molecular Chemistry, College of Industrial Technology, Nihon University, Narashino, Chiba 275-8575 Japan

## Abstract

We have developed and characterized a bacterial consortium that reductively dechlorinates trichloroethene to ethene. Quantitative PCR analysis for the 16S rRNA and reductive dehalogenase genes showed that the consortium is highly enriched with *Dehalococcoides* spp. that have two vinyl chloride reductive dehalogenase genes, *bvcA* and *vcrA*, and a trichloroethene reductive dehalogenase gene, *tceA*. The metagenome analysis of the consortium by the next generation sequencer SOLiD 3 Plus suggests that a *Dehalococcoides* sp. that is highly homologous to *D. mccartyi* 195 and equipped with *vcrA* and *tceA* exists in the consortium. We isolated this *Dehalococcoides* sp. and designated it as *D. mccartyi* UCH-ATV1. As the growth of *D. mccartyi* UCH-ATV1 is too slow under isolated conditions, we constructed a consortium by mixing *D. mccartyi* UCH-ATV1 with several other bacteria and performed metagenomic sequencing using the single molecule DNA sequencer PacBio RS II. We successfully determined the complete genome sequence of *D. mccartyi* UCH-ATV1. The strain is equipped with *vcrA* and *tceA*, but lacks *bvcA*. Comparison with tag sequences of SOLiD 3 Plus from the original consortium shows a few differences between the sequences. This suggests that a genome rearrangement of *Dehalococcoides* sp. occurred during culture.

## Introduction

Volatile chlorinated hydrocarbons, such as tetrachloroethene (PCE) and trichloroethene (TCE), are among the most abundant soil and groundwater contaminants in the world. Some microbes perform dehalorespiration, which uses chlorinated organic compounds as electron acceptors^1^. PCE and TCE are reductively dechlorinated via less-chlorinated intermediates, dichloroethene (DCE) and vinyl chloride (VC), to harmless ethene. Such *in situ* bioremediation via activation of dehalorespiration by exogenous electron donors is thought to be the most promising means of remediating soil or groundwater contaminated with chloroethenes^2^. Reductive dechlorination of PCE and TCE are performed by several bacteria species, including *Dehalococcoides* spp., but only a few *Dehalococcoides* spp. members can dechlorinate DCE and VC to ethene^3^. Supply of electron donors, such as Hydrogen Releasing Compound (HRC^®^), to soil contaminated with PCE or TCE often results in incomplete dechlorination to ethene and accumulation of DCE or VC. To avoid such problems, the administration of cultured *Dehalococcoides* spp. that can dechlorinate DCE and VC in contaminated ground, has been proposed. However, it is very difficult to isolate and culture *Dehalococcoides* spp. Among *Dehalococcoides* spp., *D. mccartyi* 195 was first reported to dechlorinate PCE to ethene^4^. Although *D. mccartyi* 195 has reductive dehalogenase (RDase) genes for PCE and TCE (*pceA* and *tceA*)^5^, it lacks VC RDase genes. Thus, VC is slowly degraded by co-metabolic pathways, and VC may accumulate. In 2003, a VC-dechlorinating enrichment culture was established, and *D. mccartyi* BAV1 was isolated as a VC-reducing bacterium^6^. In *D. mccartyi* BAV1, *bvcA* is involved in VC-reductive dechlorination^7^. *D. mccartyi* VS and FL2 have subsequently been reported to dechlorinate DCE and VC to ethene^8, 9^. These strains are equipped with another RDase gene (*vcrA*)^10^. VcrA can dechlorinate all of the isomers of DCE as well as VC^11^. To date, most *Dehalococcoides* spp. that can perform complete dechlorination to ethene have the *vcrA* gene, for example, *D.* *mccartyi* GT has *vcrA* and can dechlorinate TCE to ethene^12^. However, it is unknown how TCE is dechlorinated, as this species lacks the TCEase gene *tceA* ^5^. Recently, *D. mccartyi* UCH007 and BTF08 have been identified to possess *pceA*, *tceA* and *vcrA* and can dechlorinate of PCE or TCE to ethene^13, 14^.

*Dehalococcoides* spp. share extremely high DNA sequence homology. *Dehalococcoides* spp. are separated into three subgroups—the *Cornell*, *Victoria*, and *Pinellas* sub-branches—according to slight differences in their 16S rRNA gene sequences^15^. Recent developments in DNA sequencing technology and isolation methods enabled us to determine the genome sequences of *Dehalococcoides* spp. The genome sequences of more than ten *Dehalococcoides* spp. have been reported to date. Genome sequence similarities correlate with their classification using 16S rRNA sequences. *D. mccartyi* CG5 (CP006950) and 195 (CP000027) belong to the *Cornell* group; *D*. sp. UCH007 (CP006951), *D. mccartyi* GY50 (CP006730), VS (CP001827) and CG1 (CP006949) belong to the *Victoria* group; and *D. mccartyi* BTF08 (CP004080), BAV1 (CP000688), CBDB1 (AJ965256), GT (CP001924), DCMB5 (CP004079), CG5 (CP006951) and IBARAKI (AP014563) belong to the *Pinellas* group.

The isolation and application of *Dehalococcoides* spp. is hampered by their very slow growth rates. As *Dehalococcoides* is commonly found in microbial communities that contain other anaerobes, such as *Desulfovibrio*, *Eubacterium*, *Acetobacterium*, *Citrobacter*, *Spirochetes* and *Clostridium* ^16–19^, and it is suggested that symbiotic interactions are indispensable for the growth of *Dehalococcoides* spp. The growth of *D. mccartyi* 195 is sustained by *Desulfovibrio vulgaris* Hildenborough and *Methanobacterium congolense* ^20^.

In this study, we constructed a bacteria consortium that dechlorinates TCE to ethene and succeeded in isolating and characterizing novel *Dehalococcoides mccartyi* UCH-ATV1, which belongs to the Cornell group and possesses *tceA* and *vcrA*. Careful analysis suggests that a genome rearrangement occurred during culture.

## Results

### Construction of a bacterial consortium that can dechlorinate cis- DCE to ethene

**Figure 1:**
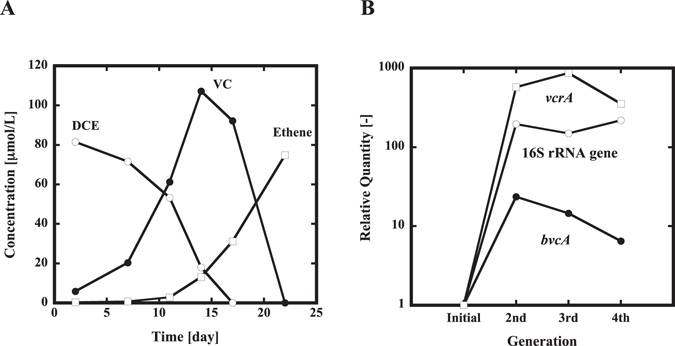
Construction of a bacterial consortium that dechlorinates *cis*-DCE to ethane. (**A**) Reductive dechlorination of *cis*-DCE to ethene by the 4^th^-generation consortium. DCE (open circle), VC (closed circle), and ethene (open square). (**B**) Increase in *Dehalococcoides* spp. in the culture-by-culture generation. Relative amounts of the *Dehalococcoides* 16S rRNA, *vcrA* and *bvcA* genes were monitored by real time qPCR. *Dehalococcoides* 16S rRNA (open circle), *bvcA* (closed circle), and vcrA (open square) genes.

Groundwater was obtained from a site contaminated with TCE and used as a source of bacteria. Dechlorination of *cis*-DCE was observed from primary culture, and bacteria responsible for *cis*-DCE dechlorination were enriched and maintained by consecutive 4% (v/v) transfers. Figure 1A shows changes in the concentration of *cis*-DCE and VC in the 4^th^ generation culture. As *cis*-DCE decreased, VC appeared, and *cis*-DCE was almost completely converted to ethene after three weeks. Similar dechlorination activity was observed in subsequent generations. Genes for the 16S rRNA of *Dehalococcoides* spp. and RDases, *vcrA* ^10^ and *bvcA* ^7^ were quantified by qPCR (Fig. 1B). The quantities of *Dehalococcoides* 16S rRNA and *vcrA* genes have increased with generation number and reached plateau. Different from them, *bvcA* increased in the beginning and then decreased gradually. The result suggests existence of two different *Dehalococcoides* spp., one with *bvcA* and the other without *bvcA*.

### Metagenome analysis of the consortium by the next generation DNA sequencer SOLiD 3 Plus

**Figure 2:**
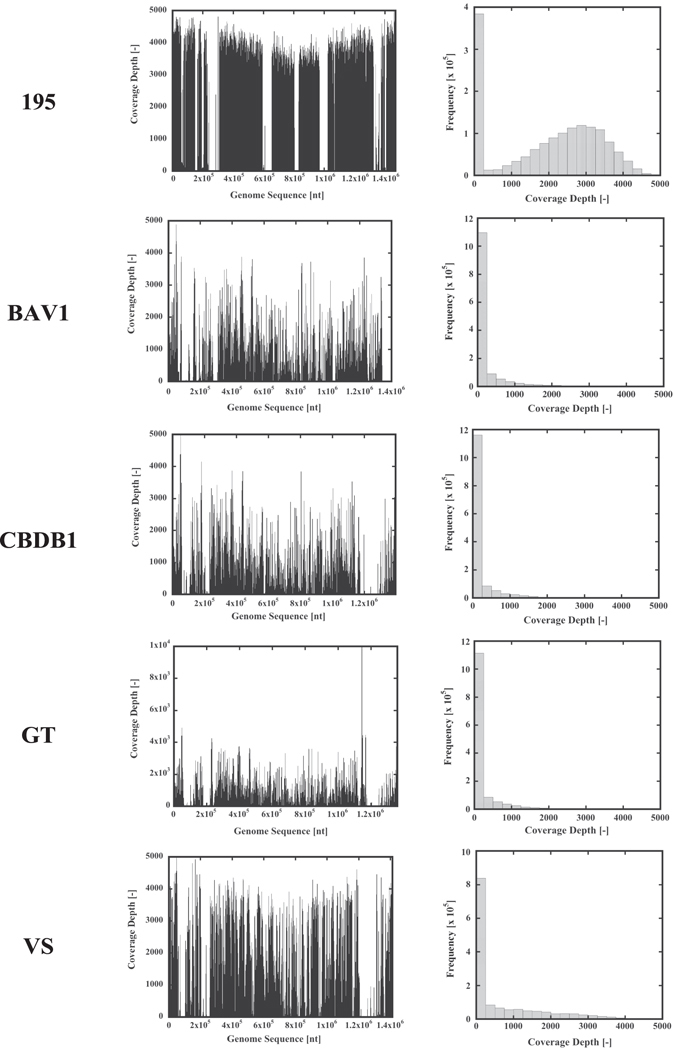
Comparison of consortium metagenome sequence data with the that of the *Dehalococcoides* sp. genome. Tag sequences for the metagenome of the consortium by SOLiD 3 Plus were matched to the genome sequences of five *Dehalococcoides* spp. (*D. mccartyi* 195, *D. mccartyi* BAV1, *D. mccartyi* CBDB1, *D. mccarty*i GT, and *D. mccartyi* VS).

As *Dehalococcoides* spp. appeared to dominate the 4^th^ generation of the bacterial consortium, the metagenome of this consortium was analyzed by the next generation DNA sequencer SOLiD 3 Plus (Thermo Fisher Scientific), and 332,003,834 reads of 50-base were obtained. Among them, 4,961,715 reads with unreliable sequences were excluded and 327,042,119 reads were used for analysis. The obtained data were compared with genome sequences of the *Dehalococcoides* strains *D*. *mccartyi* 195 (CP000027)^21^, *D. mccartyi* BAV1 (CP000688), *D. mccartyi* CBDB1 (AJ965256)^22^, *D*. *mccartyi* VS (CP001827), and *D. mccartyi* GT (CP001924). The numbers of tag sequences matched by comparison to reference genome sequences, with an allowance of two mismatches in 50 nt, coverage depths, are shown. The results clearly show that a *Dehalococcoides* sp. in the consortium are highly homologous to *D*. *mccartyi* 195 (Fig. 2).

**Figure 3:**
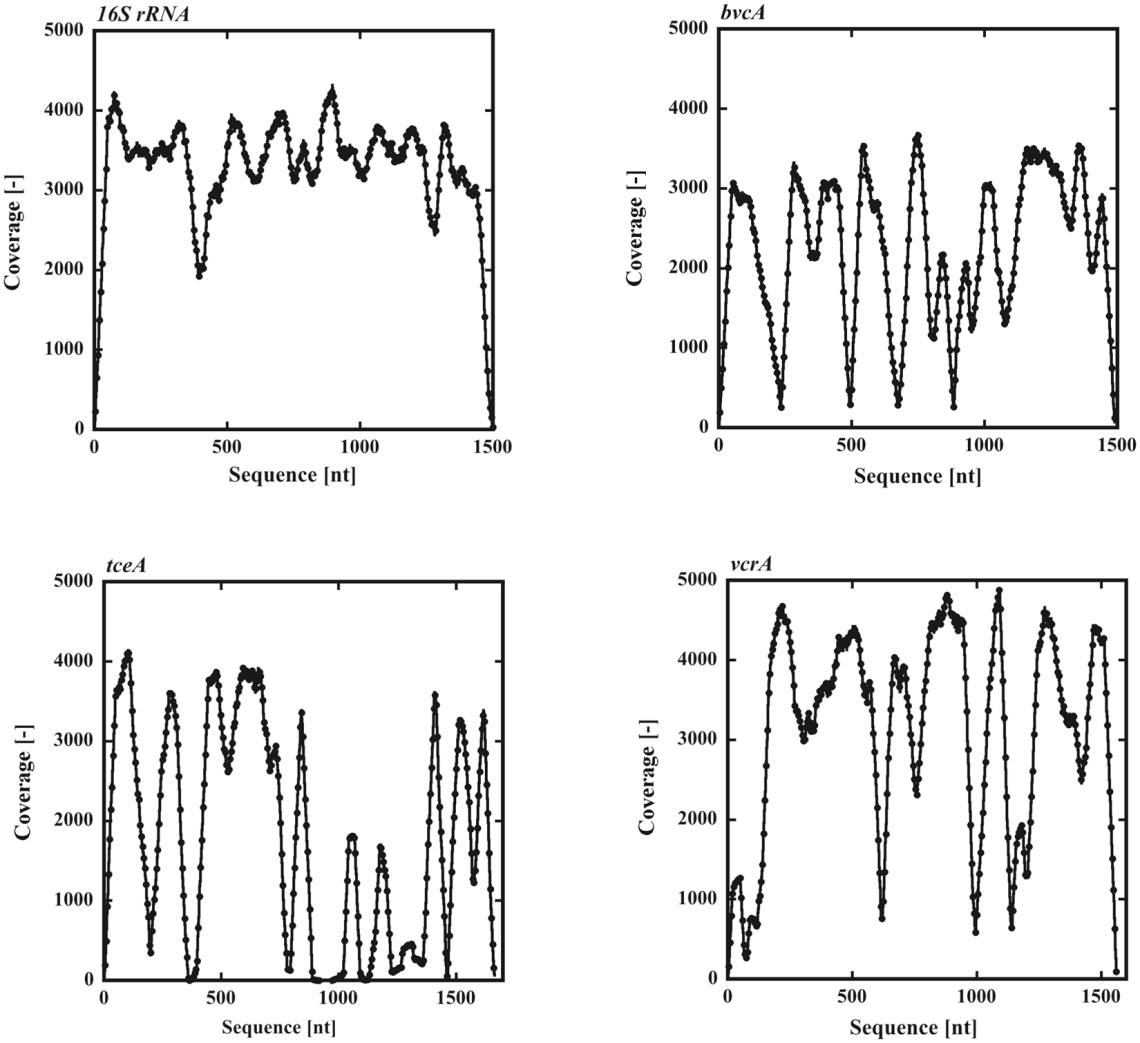
Coverage of SOLiD 3 Plus tag sequences for *Dehalococcoides* 16S rRNA and RDase genes. Tag sequences of the metagenome of the consortium by SOLiD 3 Plus were matched to sequences of *Dehalococcoides* 16S rRNA, *bvcA*, *vcrA* and *tce*A genes.

To investigate the presence of RDase genes, the average coverage depths for the RDase genes were analyzed (Supplementary Table S3). Among the 17 RDase genes in *D*. *mccartyi* 195, the presence of four genes, including TCE RDase, *tceA*, was suggested. However, the PCE RDase gene *pceA* was not detected in the metagenome of the consortium. The VC RDase genes *bvcA* of *D. mccartyi* BAV1 and *vcrA* of *D. mccartyi* VS were present with significantly high average coverages, consistent with the results of qPCR for these genes. However, the presence of other RDase genes from *D. mccartyi* BAV1 and *D. mccartyi* VS was not observed. Figure 3 shows the coverage depth of the genes for *Dehalococcoides* 16S rRNA, *tceA*, *bvcA* and *vcrA*. Reflecting the high homology of 16S rRNAs in *Dehalococcoides* spp., *Dehalococcoides* 16S rRNA exhibited a consistently high coverage depth throughout the entire gene. The coverage depth of 16S rRNA reflects the population of *Dehalococcoides* spp. In the consortium. Compared with 16S rRNA genes, the three RDase genes, *bvcA*, *tceA* and *vcrA*, showed some sequence variation. However, all exhibited significantly high identity across entire sequences. Sequence variation in *vcrA* is small compared with *bvcA* or *tceA*. Considering highly conserved sequences, the maximal coverage value should correlate with the abundance of the gene. The maximal coverage values for *Dehalococcoides* 16S rRNA, *bvcA*, *tceA* and *vcrA* are 4318, 3694, 4140 and 4878. The value for vcrA is significantly higher than those for *Dehalococcoides* 16S rRNA and *tceA*. It is reasonable that the difference is caused by variations in PCR amplification or sequence preference. Thus, we concluded that a single species *Dehalococcoides* sp. equipped with *bvcA*, *tceA* and *vcrA* dominantly existed in the consortium, and the other one without *bvcA* gradually increased. However, we don’t have solid evidence for the existence of *Dehalococcoides* sp. equipped with *bvcA*, *tceA* and *vcrA*.

The presence of *tceA* suggested that the consortium can dechlorinate TCE to ethene. The consortium was cultured in media containing 100 µM TCE to confirm this hypothesis, and this TCE was completely dechlorinated by the consortium (Supplementary Fig.S1). Therefore, we conclude that a *Dehalococcoides* sp. in the consortium can dechlorinate TCE to ethene.

We attempted to examine the bacteria comprising the consortia. Comparison against 16S rRNA gene sequences in the public database was performed as described previously^19^. We compared the obtained tag sequences with the reference sequences constructed from 357,656 bacterial 16S rRNA gene sequences. The genes were sorted according to coverage values. Homologous genes were grouped together, and the sequences that showed the highest coverage depths were selected as the representative sequences. Based on the average coverage depth, the relative populations of them were estimated assuming that each species has only one 16S rRNA gene (Table 1). *Dehalococcoides* spp. were the most abundant and were estimated to cover 47.1% of all microbes in the consortium.

**Table 1:** Population of representative bacteria constituting the original consortium.

Classification	Representative Sequence	Average Coverage	Ratio[%]
*Dehalococcoides*	DQ855129	3140.9	46.2
*Enterobacter*	AM937487	1555.9	22.9
*Azospira* (*Dechlorosoma*)	AY604956	1025.6	15.1
*Spirochaetes*	EF581152	569.1	8.4
*Bacteroidetes/Chlorobi*	AY280650	500.1	7.4

### Isolation of *D. mccartyi* UCH-ATV1

Following repeated transfers to *cis*-DCE amended media in the presence of ampicillin or 2-bromoethanesulfonate, a series of dilution-to-extinction culturing and several agar shake processes were performed, and the strain *D*. *mccartyi* UCH-ATV1 was obtained in pure culture (Supplementary Fig. S2). The presence of the RDase genes *tceA* and *vcrA* was confirmed by PCR (Supplementary Fig. S3). However, PCR amplification of *bvcA* was *in vain* (data not shown).

### Obtaining bacteria that support the growth of *Dehalococcoides* sp

**Figure 4:**
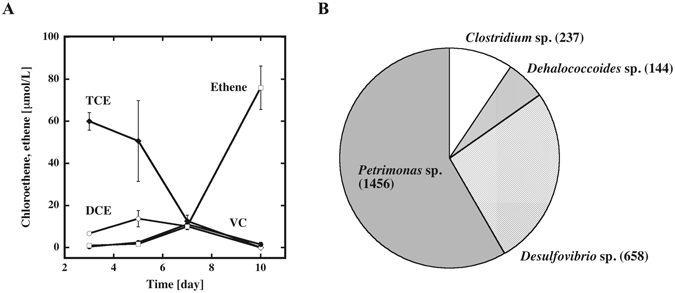
Characterization of the constructed consortium containing *D. mccartyi* UCH-ATV1. (**A**) Reductive dechlorination of TCE to ethene by the consortium. TCE (closed diamond), DCE (open circle), VC (closed circle), ethene (open square). (**B**) Classification and quantification of the 16S rRNA genes of the consortium.

Similar to other *Dehalococcoides* spp., the growth and dechlorination of the isolated *D. mccartyi* UCH-ATV1 was very slow compared with that of the consortium as a whole (Supplementary Fig. S4). Thus, we tried to obtain bacteria that support the growth of *D. mccartyi* UCH-ATV1. As the constructed consortium contained various species, we isolated *Dehalococcoides*-supporting bacteria from the consortium obtained in our previous study^19^. DGGE analysis showed that only three bacterial species including *D. mccartyi* IBARAKI dominantly existed in the Ibaraki consortium. The sequences of 16S rRNA identified the other members of the IBARAKI consortium as *Desulfovibrio desulficans* and *Eubacterium acidaminophilus*. At first, we grew the Ibaraki consortium in the absence of chloroethenes to remove *D. mccartyi* IBARAKI, and then mixed with isolated *D. mccartyi* UCH-ATV1. The prepared culture was able to convert TCE to ethene (Supplementary Fig. S5). As *D. mccartyi* IBARAKI cannot dechlorinate TCE, other bacteria in the IBARAKI consortium thus support the growth of *D. mccartyi* UCH-ATV1. Assuming that *Desulfovibrio* supports the growth of *D. mccartyi* IBARAKI, we performed single colony isolation from a shake agar culture in the media for *Desulfobivrio* species. We obtained single colonies after several rounds. However, 16S rRNA sequence analysis of the clone indicated a mixture of *Desulfovibrio* and *Petrimonas* spp. Although *Petrimonas* species were not detected by DGGE analysis in the enriched culture, they were observed in the Ibaraki consortium at an earlier stage. Thus, it is reasonable to conclude that *Petrimonas* spp. remained in the consortium and its populations increased during the single colony selection procedures. We were not able to isolate the *Desulfovibrio* species despite repeated attempts. Thus, we used a mixture of *Desulfovibrio* and *Petrimonas* spp. in culture with *D. mccartyi* UCH-ATV1. As expected, the mixed consortium exhibited significantly high dechlorination activity for TCE (Fig. 4A). Unexpectedly, when we analyzed the consortium by DGGE, six bands were observed (Supplementary Fig. S6). Among them, three major bands were identified as *Petrimonas* sp., *Dehalococcoides* ap. And *Desulfovibrio* sp. We could not clearly identify the other three bands due to poor resolution. However, only the sequences for *Dehalococcoides*, *Desulfovibrio* and *Petrimonas* species were observed in the sequences from their amplicons.

The metagenome of the consortium was subjected to sequencing by PacBio RS II. At first, PCR-amplified bacterial 16S rRNA genes were sequenced. From 14742 CCS (Circular Consensus Sequencing) reads, 2760 non-redundant sequences were obtained. Among them, 11 were chimera of two 16S rRNA sequences, and 146 were not 16S rRNA genes. Then, we compared 2525 sequences with the SILVA 123 SSURef sequences^23^. Among them 2495 sequences showed more than 99% identities with 16S rRNA sequences of *Dehalococcoides* sp. (144), *Clostridium* sp. (237), *Desulfovibrio* sp. (658) and *Petrimonas* sp. (1456) (Fig. 4B). Although remaining 130 sequence showed less than 99% identities with 16S rRNA genes in the data base. However, the most homologous 16S rRNA genes of them are 16S rRNA genes of *Dehalococcoides* sp., *Clostridium* sp., *Desulfovibrio* sp. and *Petrimonas* sp. It seems that the relatively low identity (90–98%) should be due to the mutations by PCR amplification or the sequence errors. Thus, we concluded that the consortium was composed of four species.

The unidentified weak bands in PCR-DGGE should correspond to the 16S rRNA genes from *Clostridium* sp. Generally, *Clostridium* spp. have multiple 16S rRNA genes, and we found slight sequence variations among *Clostridium* 16S rRNA genes.

### Determination of the Complete Genome Sequence of *D. mccartyi* UCH-ATV1

The metagenome of the constructed consortium was submitted to sequencing by the PacBio RS II platform with a 20-kb insert library and P6-C4 chemistry. We used 30 SMRT cells to record 240-min movies. In the first sequencing run using 16 cells, 1.4 M reads with an average length of 4.2 kb were obtained. In the second sequencing run using 14 cells, 533 K reads with an average length of 3.9 kb were obtained. We applied HGAP version 2 and version 3 to each of the sequencing data with a 6-kb Minimum Seed Read Length and a 1.4-Mb Genome Size. A total of 485 contigs was assembled, and of those, 61 contigs had similarity with *Dehalococcoides* species. We attempted to overlap and join those contigs using Minimus2, and a circular sequence was constructed. During the final circularization process with Minimus2, we achieved a complete genome sequence of a *Dehalococcoides*-like strain of 1387782 nt (Supplementary Fig. S7). The genome sequence was deposited in DDBJ with the accession number, AP017649. The number of CDS was 1498, which includes three rRNA genes (5S rRNA, 16S rRNA, 23S rRNA) and 47 tRNA genes.

**Figure 5:**
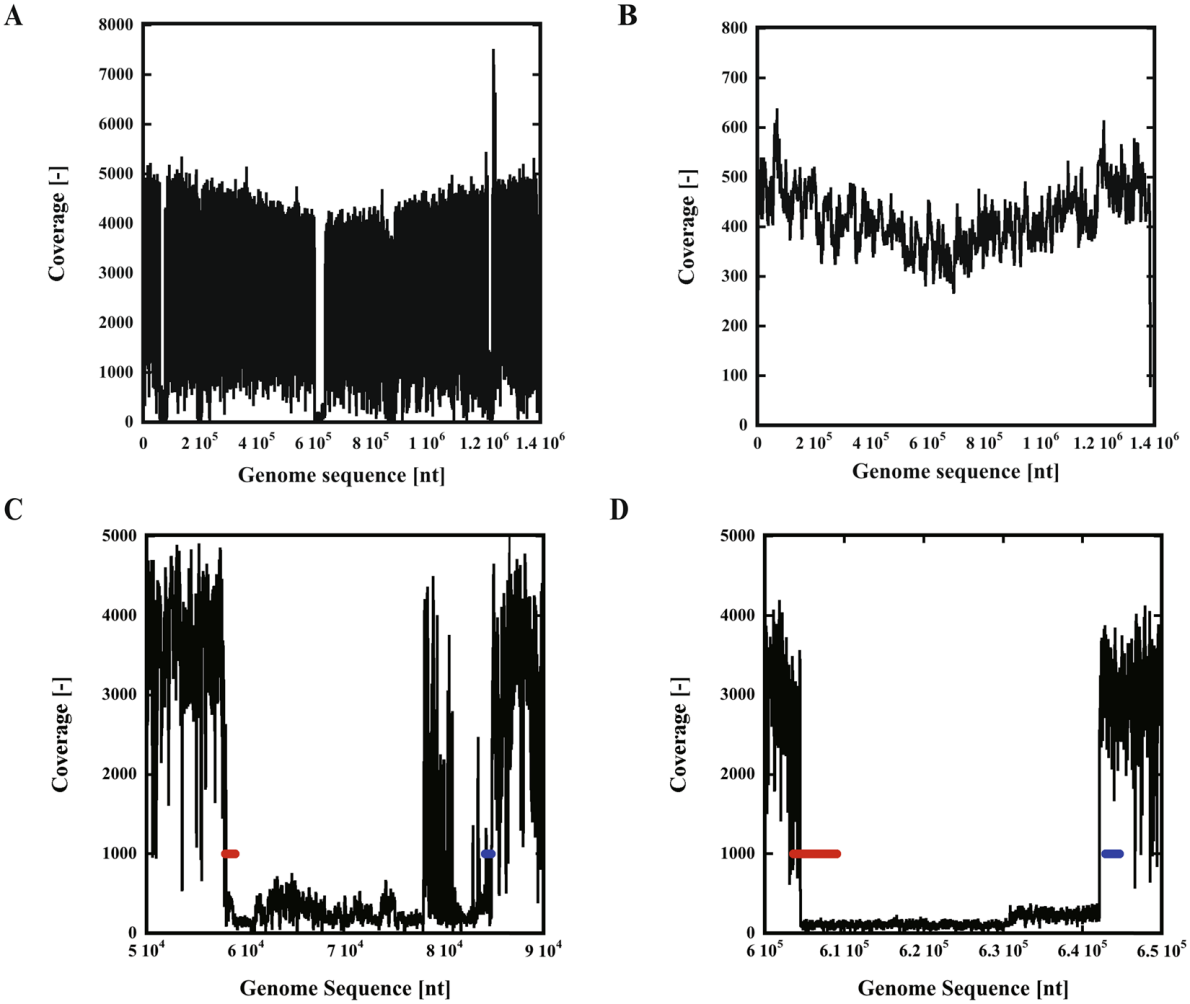
Comparison of the genome sequence of *D. mccartyi* UCH-ATV1 with the metagenome analysis data. The genome sequence of *D. mccartyi* UCH-ATV1 was compared with metagenomic data of the original consortium obtained by SOLiD 3 Plus (**A**) or the constructed consortium obtained by PacBio RS II (**B**). (**C**,**D**) Two large gap regions in comparison to the metagenome data of the original consortium (**A**) are shown. Red and blue lines represent phage- or recombinase-related genes, respectively.

To confirm that the genome actually corresponded to that of *D. mccartyi* UCH-ATV1, we matched the SOLiD 3 Plus tag sequences of the original consortium to the genome sequence (Fig. 5A). Very similar constant high-coverage values were observed throughout the genome sequence. However, several gap regions were observed where the coverage values were significantly reduced. However, PacBio RS II data matched the genome sequence in an almost uniform fashion throughout the genome (Fig. 5B).

Genome annotation also indicates that the regions do not arise from *Dehalococcoides* spp. Large gap regions are surrounded by phage recombinase-like protein-coding regions or phage-related genes (Fig. 5C and D). The red and blue lines in Fig. 5C correspond to DEHALATV1_0058 (57900 nt – 58898 nt), a site-specific recombinase phage integrase family, and DEHALATV1_0098 (84056 nt – 84679 nt), a phage-repressor-like transcriptional regulator, respectively. In Fig. 5D, the regions for DEHALATV1_0629 Restriction endonuclease S subunit-like protein, DEHALATV1_0630 SAM-dependent methyltransferase, DEHALATV1_0631 hypothetical protein, and DEHALATV1_0632 5-methylcytosine-specific restriction enzyme subunit McrC are shown as the red line, and the DEHALATV1_0669 site-specific recombinase phage integrase family is shown as a blue line.

Thus, it is reasonable to conclude that these regions originate through recombination. To confirm that they are not artifacts resulting from the contig assembly, we performed PCR amplification of the junction region between the high-coverage and low-coverage regions. We obtained a PCR product spanning the high coverage region (604436nt (coverage = 3108)) and low coverage region (609373nt (coverage = 134)) (Supplementary Fig. S8).

In addition to the genome for *D. mccartyi* UCH-ATV1, contigs for *Desulfovibrio*, *Petrimonas* and *Clostridium* species were also present, corresponding to our 16S rRNA gene analysis.

### Comparison with other *Dehalococcoides* genomes

The genome sequence was compared to the genome sequences of various *Dehalococcoides* spp. using GenomeMatcher^24^ (Supplementary Fig. S9). The results clearly show that *D. mccartyi* UCH-ATV1 has high homology with *D. mccartyi* 195.

The results of the gene annotation are summarized in Table 2. Compared with other *Dehalococcoides* spp., there is a relatively modest number of RDase genes in the genome of *D. mccartyi* UCH-ATV1 (Table 3). Eight RDase genes were full-length, and all of these, including *tceA* and *vcrA*, were found in the SOLiD 3 Plus data, but *bvcA* was lost from the genome. Most genes were accompanied by an anchor protein gene. DEHALATV1_1300 and DEHALATV1_1301 seemed to share one anchor protein gene (DEHALATV1_1302). In addition, several nonfunctional truncated RDase genes were found. Most were also accompanied by an anchor protein gene.

**Table 2:** Genome overview of *D. mccartyi* UCH-ATV1.

	D. mccartyi UCH-ATV1
Size (bp)	1387782
G + C content (%)	49
Stable RNAs	
rRNAs	3
tRNAs	47
Protein-coding sequences	1448

**Table 3:** Reductive dehalogenase genes in the genome of *D. mccartyi* UCH-ATV1.

	Functional/partial	Homology with 195 RDases	Homologous RDases	Anchoring protein
DEHALATV1_0101	partial			
DEHALATV1_0166		DET0180/100%	WP_010935983/100%	DEHALATV1_0167
DEHALATV1_0203	partial			DEHALATV1_0205
DEHALATV1_0204	partial			DEHALATV1_0205
DEHALATV1_0909	*tceA*	DET0079/97%	WP_015407437/100%	DEHALATV1_0908
DEHALATV1_1275	*vcrA*	—	WP_061977500/100%	DEHALATV1_1274
DEHALATV1_1288	partial			
DEHALATV1_1300		—	WP_058292018/100%	DEHALATV1_1302
DEHALATV1_1301		—	WP_058292017/100%	DEHALATV1_1302
DEHALATV1_1335	partial			DEHALATV1_1339
DEHALATV1_1342		DET1522/84%	WP_058291931/100%	DEHALATV1_1341
DEHALATV1_1346		DET1528/100%	WP_010937203/100%	DEHALATV1_1345
DEHALATV1_1351	partial			DEHALATV1_1350
DEHALATV1_1367		DET1545/99%	WP_010937220/100%	DEHALATV1_1366

## Discussion

In this study, we developed a bacterial consortium that reductively dechlorinates *cis*-DCE and vinyl chloride VC to ethene from groundwater contaminated with trichloroethene TCE. To characterize the *Dehalococcoides* spp. and other bacterial members, genome sequencing was used to deduce the metagenome of the consortium using the next generation sequencer SOLiD 3 Plus. The genome sequence of the *Dehalococcoides* spp. was highly homologous to that of *D. mccartyi* 195 across the total genome. Interestingly, two known RDase genes for VC, *bvcA* and *vcrA*, were found in addition to the TCE RDase gene, *tceA*. Results of quantitative PCR for the RDase genes suggested existence of two difference of *Dehalococcoides* spp., one with *bvcA* and the other without *bvcA at the fourth generation*. The quantification of genes for *Dehalococcoides* 16S rRNA and RDases from the metagenomic data by SOLiD 3 Plus has suggested existence of a *Dehalococcoides* sp. equipped with three RDase genes, *tceA*, *vcrA* and *bvcA*. Although there is no solid evidence for the existence of such *Dehalococcoides* sp., the idea is supported by fact that the genome sequence of *D. mccartyi* UCH-ATV1 retains the trace of genome rearrangement.

We next isolated a *Dehalococcoides* sp. We designated it *D. mccartyi* UCH-ATV1. Counter to our expectation, it contained *tceA* and *vcrA*, but lacked *bvcA*. Further study of *D. mccartyi* UCH-ATV1 was hampered by its slow growth rate. We tried to enhance the growth of *D. mccartyi* UCH-ATV1 by mixing it with several bacteria spp. As *Desulfovibrio* sp. is thought to support the growth of *Dehalococcoides* spp.^20^, we tried to isolate a *Desulfovibrio* sp. from the consortium that was obtained in our previous studies. In the shake agar culture, we isolated black colonies, reflecting the production of sulfides by *Desulfovibrio* sp. Although we repeated the isolation process several times, only a *Petrimonas* sp. was obtained. As expected, the mixed consortium exhibited high dechlorination activity on TCE. DGGE and metagenome analysis indicated that the consortium is composed of *Desulfovibrio* sp., *Petrimonas* sp., and *Clostridium* sp. in addition to *D. mccartyi* UCH-ATV1. We have assembled the draft genome sequence of *Clostridium* sp. in the constructed consortium. The genome sequence matched well with the metagenome data of Ibaraki consortium almost throughout the genome (Supplementary Fig. S10). On the contrary, it does not match with the metagenome data of the original consortium containing *D. mccartyi* UCH-ATV1 (Supplementary Fig. S10). We observed almost same results for *Petrimonas* sp. and *Desulfovibrio* sp. Consequently, only the genome sequence of *D. mccartyi* UCH-ATV1 retains the traces of the original metagenome data. Therefore, we can conclude that *D. mccartyi* UCH-ATV1 was isolated before mixing with the bacteria from Ibaraki consortium.

Finally, we determined the complete genome sequence of *D. mccartyi* UCH-ATV1. Reflecting previous results, there was significantly high sequence identity with *D. mccartyi* 195 throughout the genome, which was equipped with *tceA* and *vcrA*. Eight full-length RDase genes and seven nonfunctional truncated RDase genes were detected. The presence of nonfunctional truncated RDase genes has also been reported in the genomes of other *Dehalococcoides* spp.^25^. Thirty-eight orthologous RDase genes were identified in *D. mccartyi* MB, of which four (DehaMB_1_0001, DehaMB_1_0161, DehaMB_1_0162, and DehaMB_8_0246) were partial sequences. Eleven RDase genes were identified in *D. mccartyi* 11a, of which Deha11a_2_0134 (281 bp) and Deha11a_6_0020 (542 bp) were truncated sequences. Surprisingly, the genome sequence differs from the metagenome analysis data of the initial consortium. As *D. mccartyi* UCH-ATV1 originated from the consortium, the genome must completely match the tag sequences of the metagenome obtained by SOLiD 3 Plus. We suspect that the difference is caused by chimera formation during the contig assembly process. However, this was ruled out by both the uniform coverage values throughout the genome and by PCR amplification of the suspicious region. In addition, the GC content values of the gap regions clearly different from those of the high coverage regions (Supplementary Fig. S11). Since the low-coverage regions are surrounded by phage-related genes or recombinase genes, we conclude that the difference is due to genome rearrangement by recombination during isolation. We hypothesize that the disappearance of the *bvcA* gene might be due to such recombination. Although further study is needed to confirm this hypothesis, it is plausible, as genome rearrangement frequently occurs in *Dehalococcoides* spp. Genome-wide studies of *Dehalococcoides* spp. will be required to confirm this hypothesis.

## Methods

### Chemicals and materials

TCE, *cis*-DCE and VC were purchased from Wako Pure Chemical Ind. Ltd. (Osaka, Japan). Ethene and H_2_ were obtained from GL Sciences Inc. (Tokyo, Japan). Sediment mud used for culture was obtained from a lotus field and sterilized by autoclave before use. All other chemicals used were reagent-grade or higher unless otherwise specified.

### Analytical methods

The concentrations of chloroethenes and ethene were determined using a Shimadzu GC 1024 gas chromatograph (Shimadzu Co., Kyoto, Japan) equipped with a DB-624 column (60 m length, 0.32 mm diameter, 1.80 μm film thickness, Agilent Technology, Santa Clara, CA, USA) and a flame ionization detector by injecting 100 μl of reactor headspace using a gas-tight syringe.

### Culture medium

The composition of the culture medium was as follows: 10 (ml/L) salt stock solution, 1 (ml/L) trace element A solution, 1 (ml/L) trace element B solution, 10 (ml/L) vitamin solution, 0.68 (g/L) sodium acetate, 2.52 (g/L) sodium bicarbonate, 1 (mg/L) resazurin-Na, 10 (ml/L) reducing agent solution and appropriate quantities of TCE or *cis*-DCE. Ingredients except for vitamin, reducing agents and chlorinated ethylenes were mixed, and the medium was dispensed into suitable culture vessels under a stream of H_2_/CO_2_ (80/20), sealed with Teflon-coated butyl rubber stoppers, and autoclaved at 121 °C for 20 min. After autoclaving, vitamin, reducing agents and chlorinated ethylenes were aseptically and anaerobically added to the respective vessels. The contents of the salt stock solution, trace element A, trace element B and reducing agent solution are described in Supplementary Table S1.

### Enrichment and cultivation

Groundwater was obtained from a site contaminated with TCE and used as a source of bacteria. The first-stage enrichment culture was carried out as follows: 4.5 g of sediment mud and 86.4 ml of the medium were added to 100-ml glass vials. The vials were sealed with Teflon-coated butyl rubber caps and crimped with aluminum rings. Vials were purged by injecting nitrogen gas for 3 min and were autoclaved at 121 °C for 15 min. After cooling, 3.6 ml of ground water was added, 5 ml of headspace gas was withdrawn from each bottle using a syringe, and 5 ml of hydrogen was then injected. Finally, *cis*-DCE was added to an initial concentration of 100 µM, and the cultures were incubated at 21 °C without shaking in the dark. After nearly all *cis*-DCE was dechlorinated, 3.6 ml of the culture was used for a successive culture in the same manner. The culture was maintained in media supplemented with TCE.

### DNA extraction

DNA was extracted from 0.5 ml of enrichment culture using the ISOIL for Beads Beating Kit (Nippon Gene Co. Ltd., Tokyo, Japan) according to the manufacturer’s instructions. The extracted DNA was suspended in 100 μl of TE buffer (pH 8.0). DNA extracts were stored at −20 °C for further analysis.

### Quantitative PCR (qPCR) for *Dehalococcoides* 16S rRNA gene and dehalogenase genes (tceA, bvcA, vcrA)

The *Dehalococcoides* 16S rRNA gene and dehalogenase genes (*tceA*, *bvcA*, *vcrA*) were quantified by qPCR using a StepOne™ real time PCR system (Thermo Fisher Scientific, Waltham, MA). The quantities of the *Dehalococcoides* 16S rRNA, *tceA* and *bvcA* genes were analyzed using a TaqMan™ method with TaqMan™ Universal PCR Master Mix (Thermo Fisher Scientific) following the supplier’s instruction. *VcrA* was quantified using a SYBR™ Green Dye method and Fast SYBR™ Green Master Mix (Thermo Fisher Scientific). The sequences of primers and probes are shown in Supplementary Table S2.

### Denaturing Gradient Gel Electrophoresis (DGGE)

PCR amplification was conducted using the primers 341fGC (5′- CGC CCG CCG CGC GCG GCG GGC GGG GCG GGG GCA CGG GGG GCC TAC GGG AGG CAG CAG -3′) and 534r (5′-ATT ACC GCG GCT GCT GC-3′) to amplify fragments of 16S rRNA genes. The PCR cycling conditions were as follows: initial denaturation at 95 °C for 10 min; eight cycles (95 °C for 1 min, annealing at 64 °C for 1 min, and extension at 72 °C for 2 min); 30 cycles (95 °C for 1 min, annealing at 55 °C for 1 min, and extension at 72 °C for 2 min); and a final extension at 72 °C for 10 min. PCR amplification reactions were carried out as described previously. DGGE was performed according to a modification of the described methods. We used 10% polyacrylamide gels with a urea-formamide denaturant gradient of 40–60%, and gels were run on a DCode Universal Mutation Detection system (Bio-Rad) for 5 h at 60 °C and 130 V. After electrophoresis, the gels were stained with SYBR Green (TAKARA BIO Ink, Shiga, Japan). Prominent bands were excised and dissolved in 200 μl of TE buffer. Each target fragment of DNA was recovered by ethanol precipitation and amplified using the primer sets 341fGC and 534r. The PCR cycling conditions were as described above. The PCR products were purified and sequenced as described previously^19^. The PCR products were purified using the Wizard SV Gel and PCR Clean-Up System (Promega). Each DNA fragment was subcloned into a plasmid by TA cloning and submitted for DNA sequencing. Sequencing reactions were run according to manufacturer’s instructions for the ABI Big Dye Terminator KIT version 3.1 (Thermo Fisher Scientific). Sequence analysis was performed using a 3130xl Genetic Analyzer (Thermo Fisher Scientific). The similarities between the obtained DNA sequences were analyzed using BLAST.

### Metagenomic Sequencing by SOLiD 3 Plus

Fragment libraries were constructed according to manufacturer’s instructions using the SOLiD™ Fragment Library Construction Kit and sequenced on a single plate of an ABI SOLiD 3 Plus sequencer (Thermo Fisher Scientific) to generate 50-nt reads. The short reads were mapped to the genome sequences of various *Dehalococcoides* spp. and reference DNA constructed from 16S rRNA gene sequences in color space using Corona-Lite or Bowtie^26^. The coverage values were calculated from Bowtie reads obtained using mpileup in SAMTools. The average coverage was calculated by averaging the coverage values for the gene or whole genome.

### Metagenomic Assembly by PacBio RS II

SMRTbell libraries were constructed according to manufacturer’s instructions using the Greater Than 10 kb Template Preparation Using AMPure PB Beads and Sequencing (MagBead Station) protocols (Pacific Biosciences, Menlo Park, CA, USA). The extracted DNA was purified using a PowerClean DNA Clean-Up Kit (MoBio laboratories, Carlsbad, CA, USA). Small fragments of DNA were removed using AMPure PB Beads (Pacific Biosciences) of 0.45x. SMRTbell 20-kb libraries were prepared using a SMRTbell Template Prep Kit 1.0 (Pacific Biosciences). Libraries were subsequently sequenced on PacBio RS II (Pacific Biosciences) using DNA/Polymerase Binding Kit P6 v2 (Pacific Biosciences) and a DNA Sequencing Reagent Kit 4.0 (Pacific Biosciences). The titration density was 0.025 nM. The template was loaded into SMRT Cell v3 (Pacific Biosciences) using a Mag Bead Kit (Pacific Biosciences). Sequencing was performed using 30 cells in total. A 240-min movie was recorded for each cell. Metagenomic assembly was performed using a Hierarchical Genome Assembly Process (HGAP)^27^. Minimus2 was used to attempt to overlap and join the resulting contigs^28^. Final assemblies were achieved by polishing the draft assemblies using Quiver^27^.

## Electronic supplementary material

Supplementary Information
