# Supplementary material for: Isolation and genomic characterization of a Dehalococcoides strain suggests genomic rearrangement during culture
Source: Sci Rep. 2017 May 22;7:2230. doi: 10.1038/s41598-017-02381-0 (PMC5440377; doi:10.1038/s41598-017-02381-0)
Supplement: Supplementary file 1 — Supplementary Information [file 41598_2017_2381_MOESM1_ESM.pdf]

Isolation and genomic characterization of a *Dehalococcoides* strain suggests genomic rearrangement during culture

Masafumi Yohda[1*], Kentaro Ikegami[1], Yuto Aita[1], Mizuki Kitajima[1], Ayane Takechi[1], Megumi Iwamoto[2], Tomomi Fukuda[2], Noriyoshi Tamura[2], Junji Shibasaki[3], Seiji Koike[3], Daisuke Komatsu[4], Sakari Miyagi[4], Minoru Nishimura[4], Yoshihito Uchino[5], Akino Shiroma[6], Makiko Shimoji[6], Hinako Tamotsu[6], Noriko Ashimine[6], Misuzu Shinzato[6], Shun Ohki[6], Kazuma Nakano[6], Kuniko Teruya[6], Kazuhito Satou[6], Takashi Hirano[6], Osami Yagi[7]

[1] Department of Biotechnology and Life Science, Tokyo University of Agriculture and Technology, Koganei, Tokyo 184-8588, Japan

[2] PaGE Science, Koganei, Tokyo 184-8588, Japan

[3] ADEKA Co., Ltd., Arakawa, Tokyo 116-8554, Japan,

[4] In Situ Solutions, Co., Ltd., Chiyoda, Tokyo 101-0044, Japan

[5] Biological Resource Center, National Institute of Technology and Evaluation, Shibuya, Tokyo 151-0066, Japan

[6] Okinawa Institute of Advanced Sciences, Uruma, Okinawa, Japan

[7] Department of Applied Molecular Chemistry, College of Industrial Technology, Nihon University, Narashino, Chiba 275-8575, Japan

*Corresponding author: Masafumi Yohda: yohda@cc.tuat.ac.jp

Running title: Genomic characterization of a *Dehalococcoides* strain

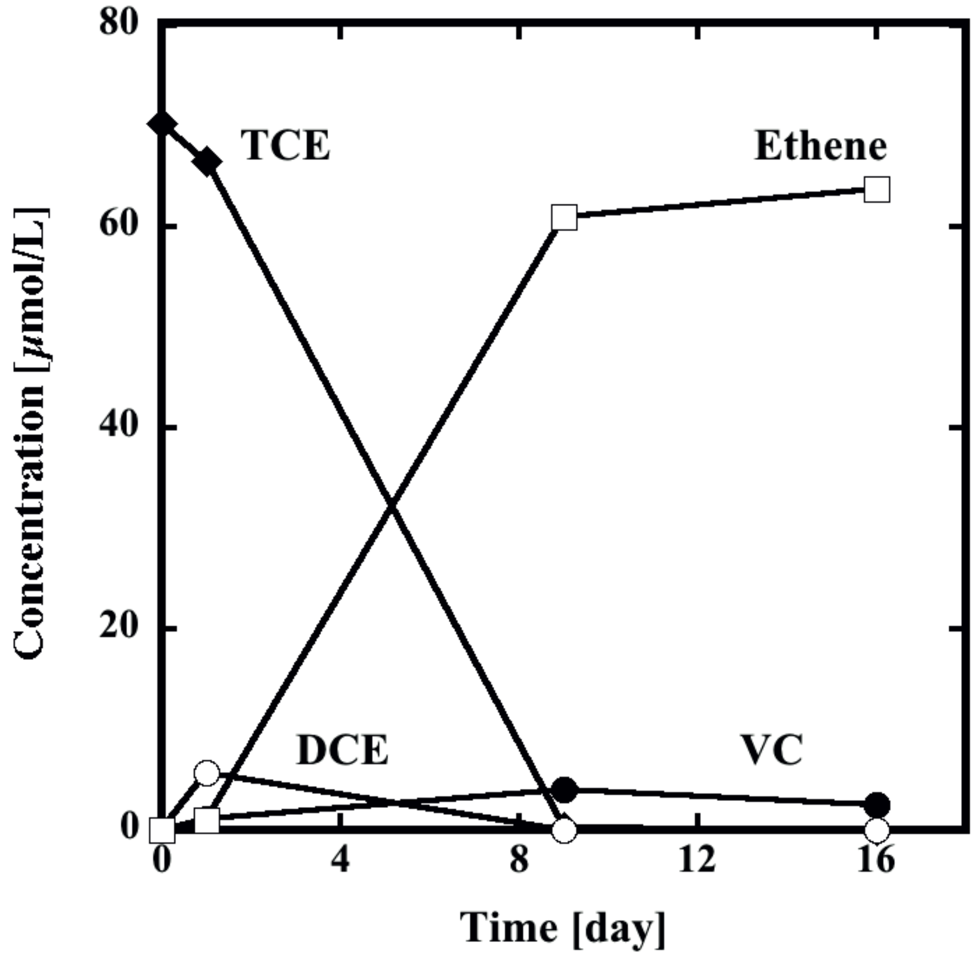

Supplementary Fig.S1
Reductive dechlorination of TCE to ethene by the original consortium.
TCE (closed diamond), DCE(open circle), VC(closed circle), ethene (open square)

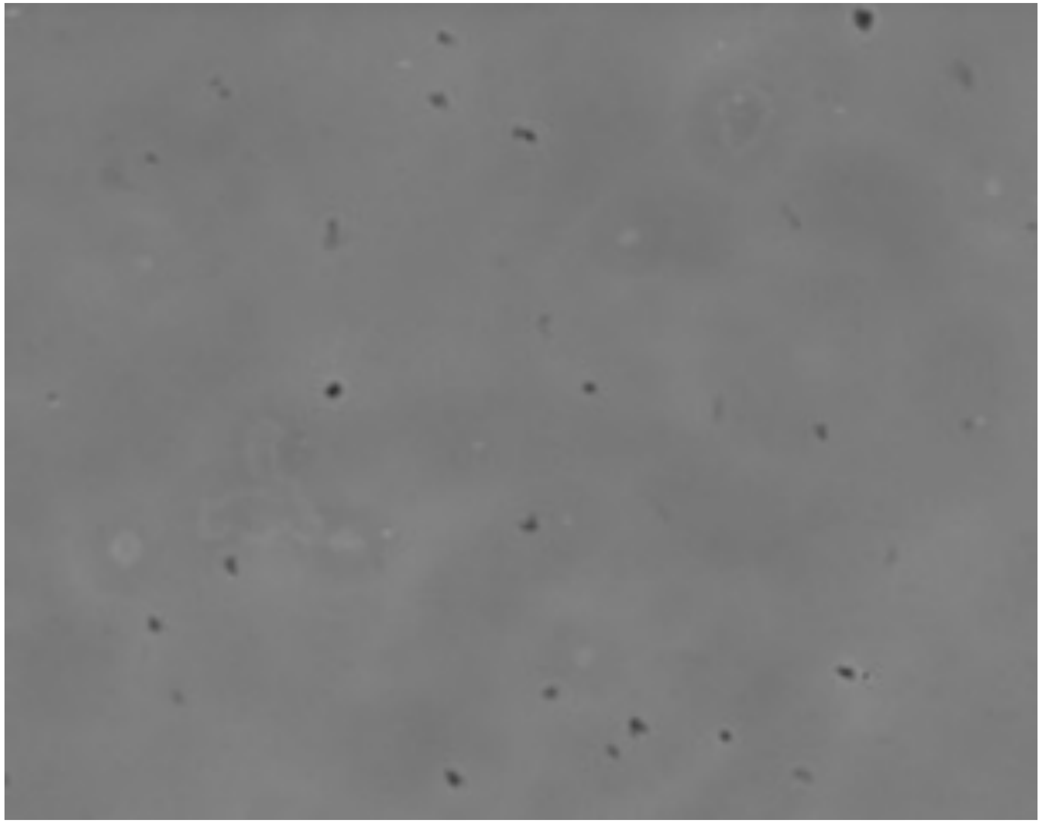

Supplementary Fig. S2
Phase contrast microscopic image of colonies for *D. mccartyi* UCH-ATV1

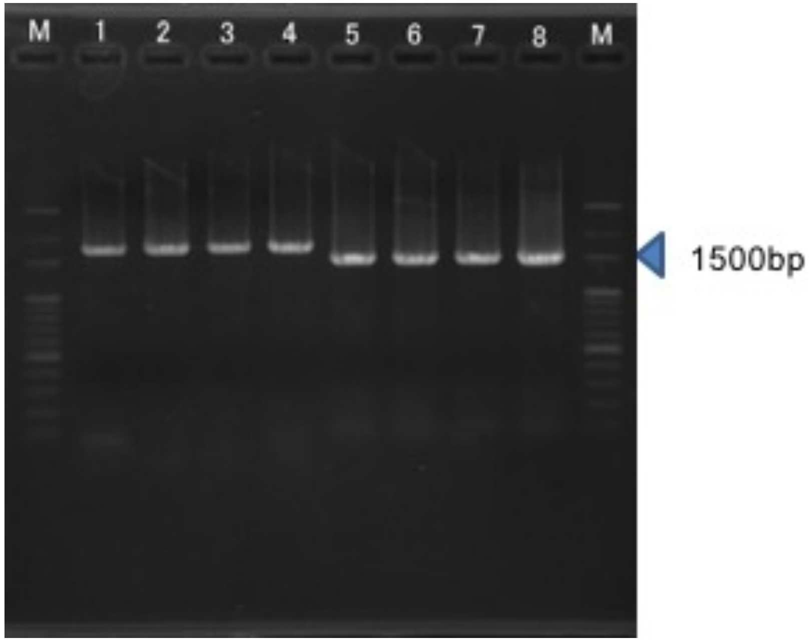

Supplementary Fig. S3　PCR amplification of *tceA* and *vcrA* of the isolated colonies

Lane M. 100bp ladder marker; Lane 1. tceA colony 1; Lane 2. tceA colony 2; Lane 3. tceA colony 3; Lane 4. tceA colony 4; Lane 5. vcrA colony 1; Lane 6. vcrA colony 2; Lane 7. vcrA colony 3; Lane 8. vcrA colony 4

Primer set for tceA （product length　1732bp.)
tceA_797F　　　ACGCCAAAGTGCGAAAAGC
tceA_2490R　　　TAATCTATTCCATCCTTTCTC
Primer set for vcrA （product length　1482bp.)
vcrA_F1 CTATGAAGGCCCTCCAGATGC
vcrA_R1 GTAACAGCCCCAATATGCCAAGTA

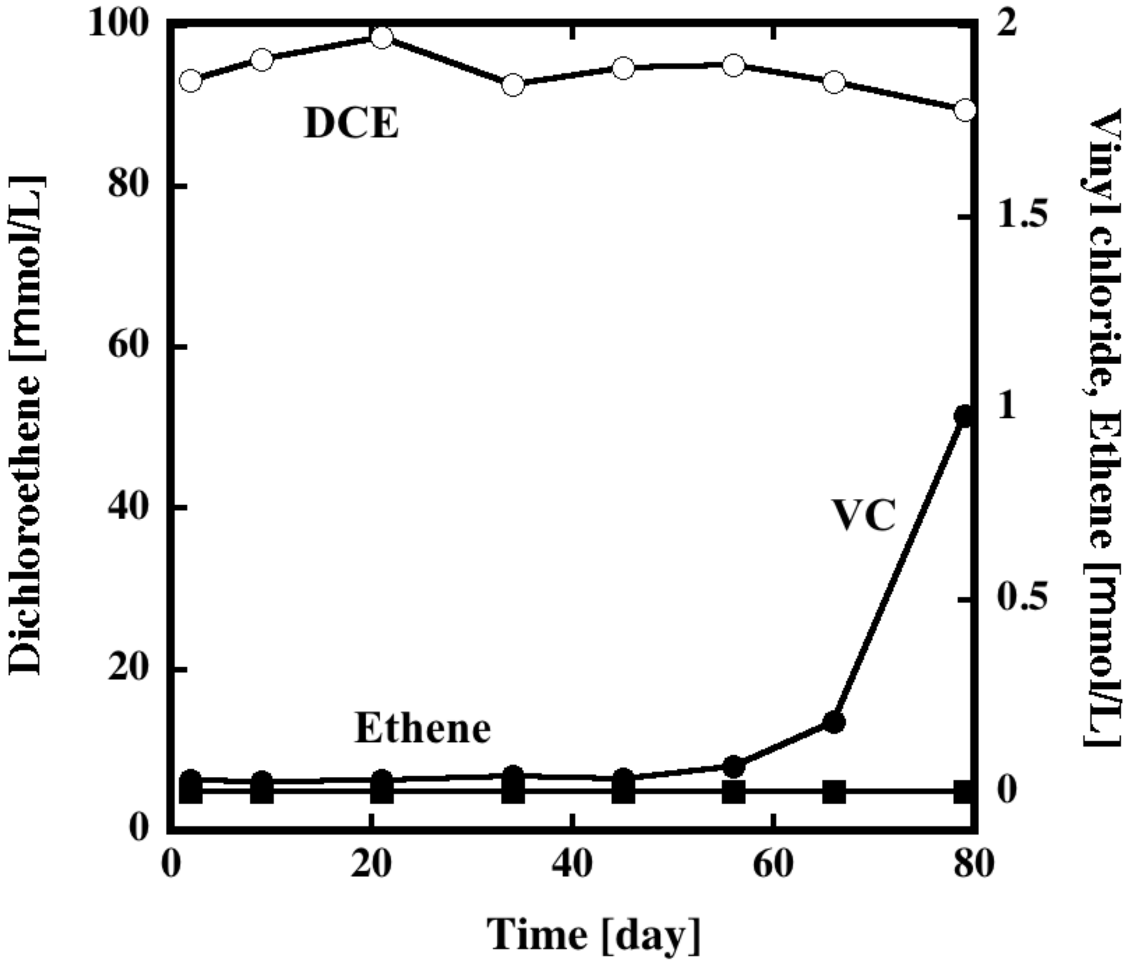

Supplementary Fig. S4
Reductive dechlorination of DCE by D. mccartyi UCH-ATV1
DCE(open circle), VC(closed circle), ethene (open square)

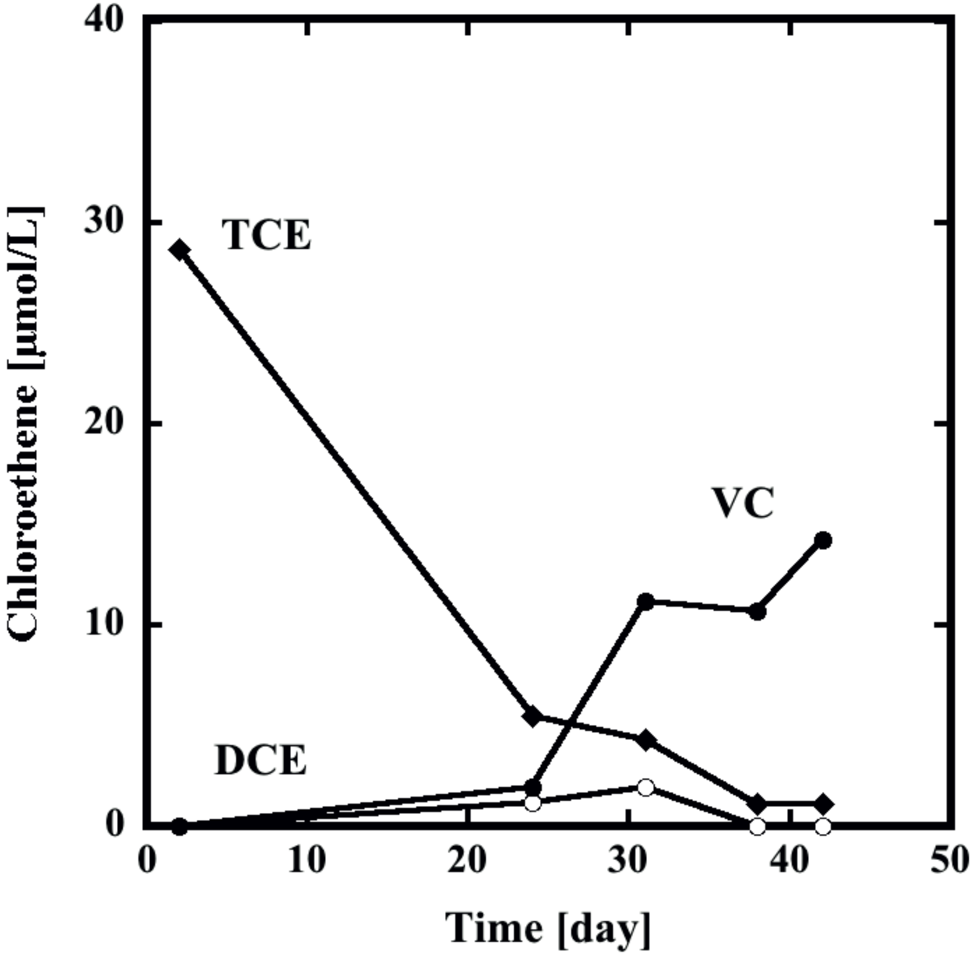

Supplementary Fig. S5
Reductive dechlorination of TCE to ethene by mixed culture of D. mccartyi UCH-ATV1 and microbes in the consortium obtained in the previous research
TCE (closed diamond), DCE (open circle), VC (closed circle)

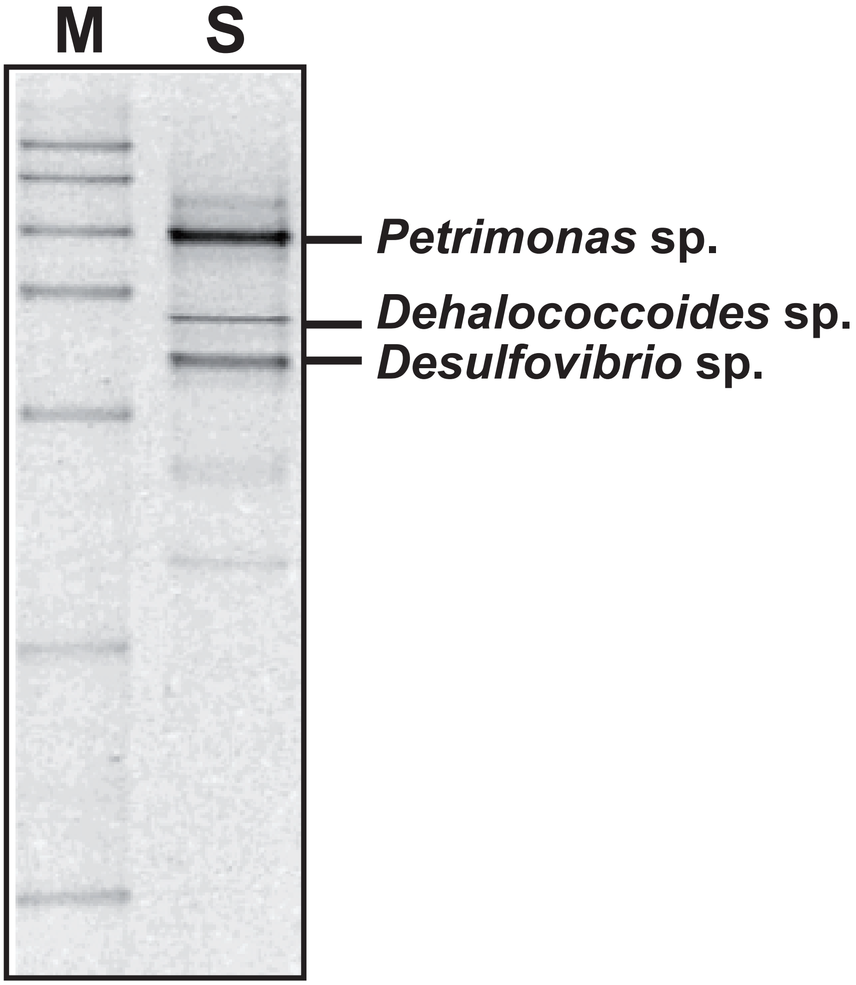

Supplementary Fig. S6

DGGE analysis of 16S rRNA genes in the constructed consortium

(M) Marker, (S) sample

Three major bands were identified as the 16S rRNA genes of *Petrimonas* sp. *Dehalococcus* sp. and *Desulfovibrio* sp.

Supplementary Fig. S7
Genome assembly of D. mccartyi UCH-ATV1

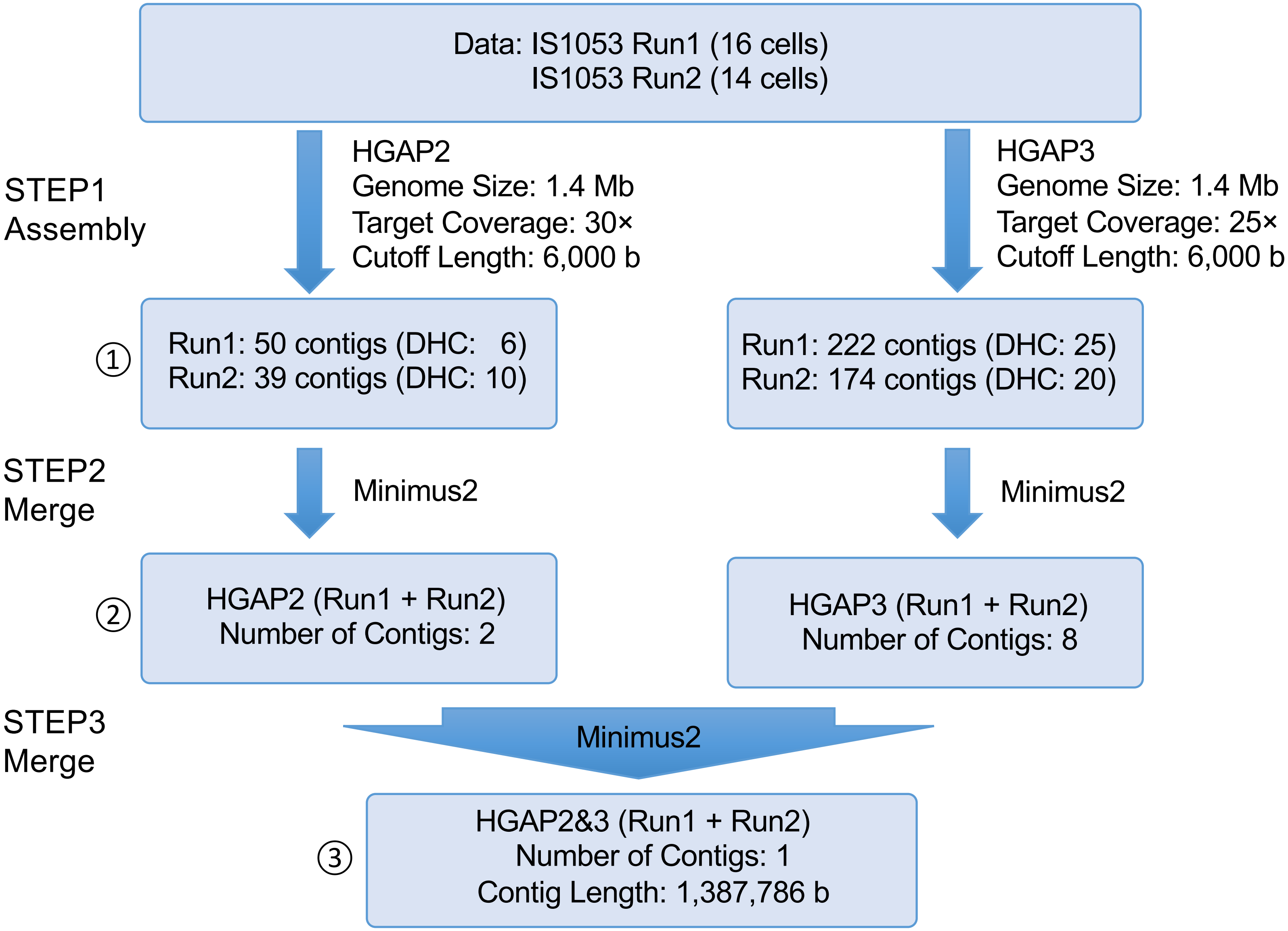

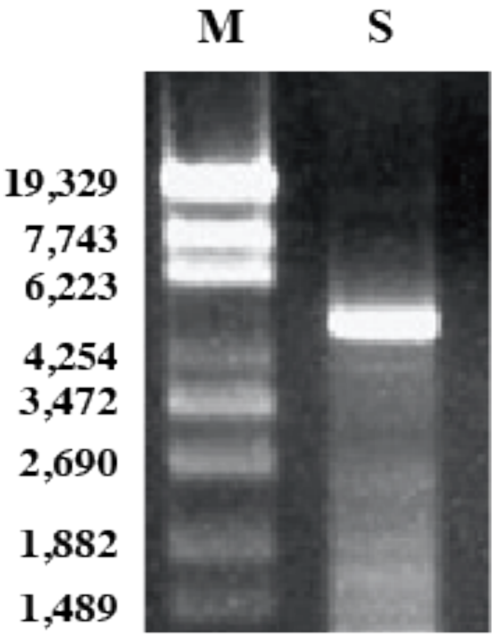

Supplementary Fig. S8 PCR amplification of the gap region

PCR amplification was performed to covering from the high coverage region (604436nt (coverage = 3108)) to the low coverage region (609373nt (coverage = 134). The expected size of PCR product is 4937 bp.

Primers used are 5’-CCCTTACAGTAATTGGCCGTTC-3’ and 5’-CTGGTACGAATACGTCGAATACGG-3’.

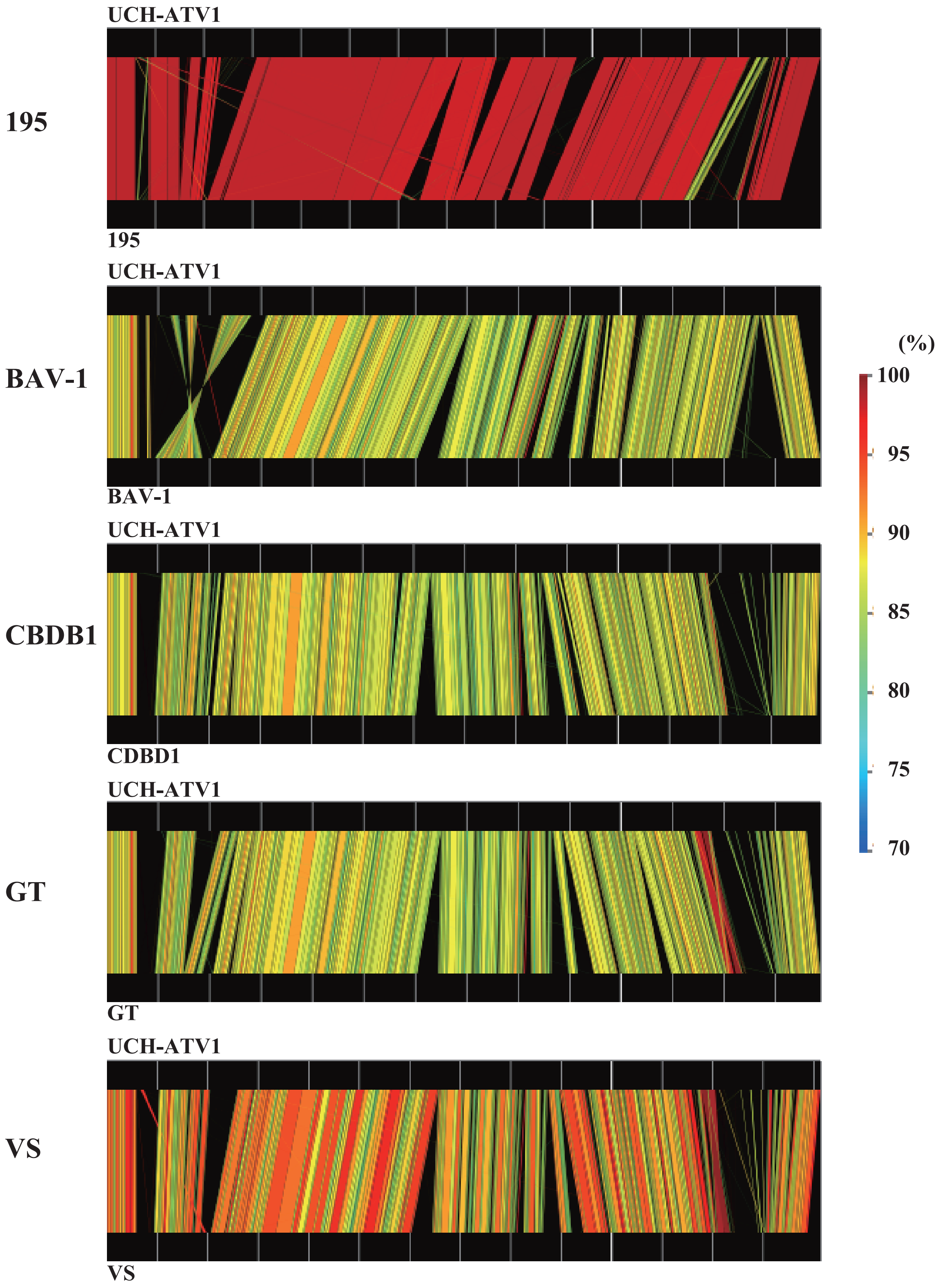

Supplementary Fig. S9 Comparison of *D. mccartyi* UCH-ATV1 genome with genome sequences of 5 *Dehalococcoides* spp.

D. mccartyi UCH-ATV1 genome was compared with genomes of *D. maccartyi* 195, *D. maccartyi* BAV1, *D. maccartyi* CBDB1, *D. maccartyi* GT, and *D. maccartyi* VS by GenomeMatcher.

Supplementary Fig. S10
Comparison of the draft genome sequence of *Clostridium* sp. with metagenome sequence data.

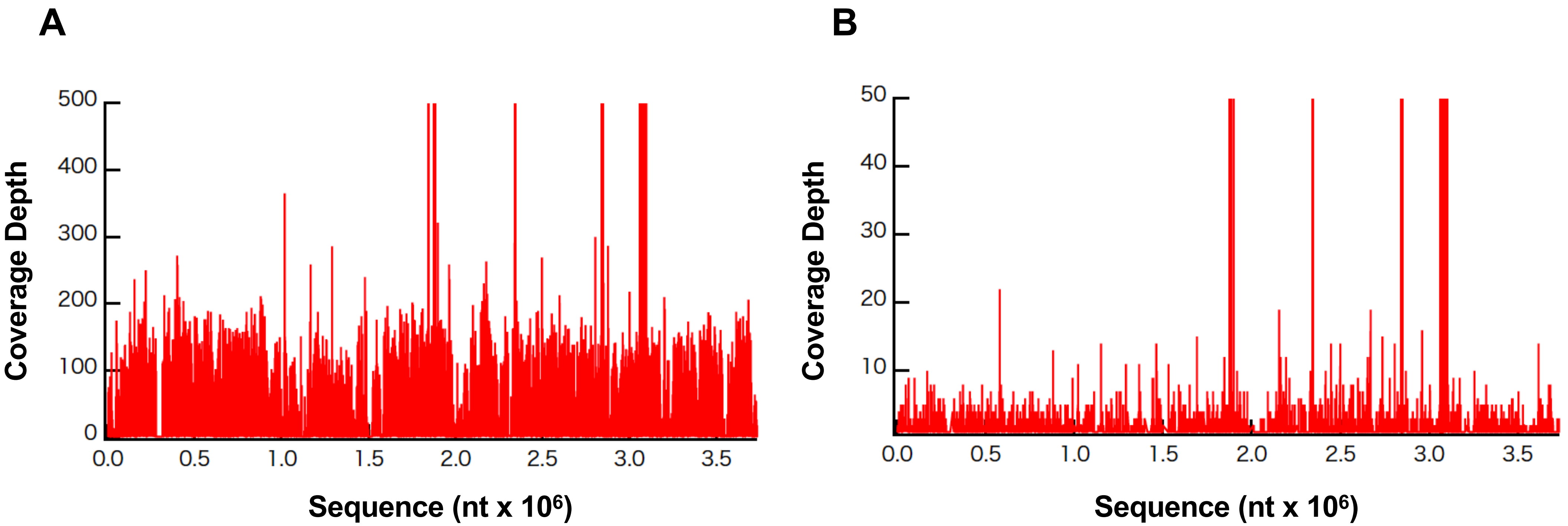

(A) Comparison with the metagenome data of the Ibaraki consortium
The ratio of sequences with the coverage depth of 0 is 28.4%
(B) Comparison with the metagenome data of the original consortium
The ratio of sequences with the coverage depth of 0 is 93.0 %

Supplementary Fig. S11
GC content of the genome of *D. mccartyi* UCH-ATV1

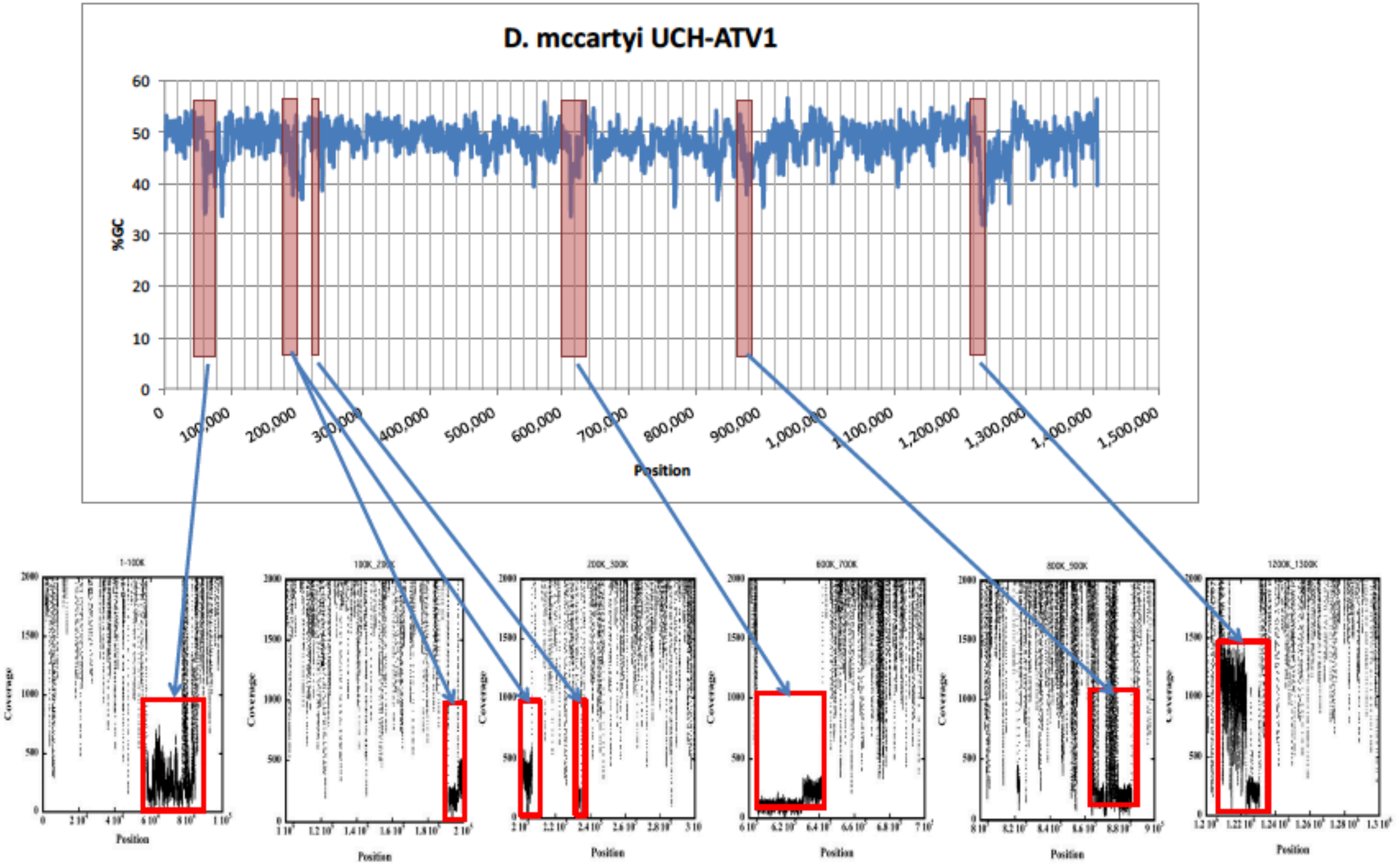

Supplementary Table S1 Stock Solution and Trace elements

100 x Salt Stock Solution

Chemical	Concentration
NaCl	100 g/L
$MgCl_2 \cdot 6H_2O$	50 g/L
KH_2PO_4	20 g/L
NH_4Cl	30 g/L
KCl	30 g/L
$CaCl_2 \cdot 2H_2O$	1.5 g/L

1000 x Trace Element A

Chemical	Concentration
HCl (25% solution w/w)	1% (v/v)
$FeCl_2 \cdot 4H_2O$	1.5 g/L
$CoCl_2 \cdot 6H_2O$	190 mg/L
$MnCl_2 \cdot 4H_2O$	100 mg/L
$ZnCL_2$	70 mg/L
H_3BO_3	6 mg/L
$Na_2MO_4 \cdot 2H_2O$	36 mg/L
$NiCl_2 \cdot 6H_2O$	24 mg/L
$CuCl_2 \cdot 2H_2O$	2 mg/L

1000 x Trace Element B

Chemical	Concentration
$NaSeO_3 \cdot 5H_2O$	6 mg/L
$Na_2WO_4 \cdot 2H_2O$	8 mg/L
NaOH	500 mg/L

Supplementary Table S2 Primers for quantitative PCR

Primer Name	Sequence
RT16SDhcF	5’-CTGGAGCTAATCCCCAAAGCT-3’
RT16SDhcR	5’-CAACTTCATGCAGCCGGG-3’
RT16SDHCP	5’-FAM-CCTCAGTCCGGATTGC-MGB-3’
tceA1270F	5’-ATCCAGATTATGACCCTGGTGAA-3’
tceA1336R	5’-GCGGCATATATTAGGGCATCTT-3’
tceAProbe	5’-FAM-CTATGGCGACCGCAGG-MGB-3’
bvcA925F	5’-AAAAGCACTTGGCTATCAAGGAC-3’
bvcA1017R	5’-CCAAAAGCACCACCAGGTC-3’
bvcA977Probe	5’-FAM-CGACGTGGCTATGTGG-MGB-3’
vcrA1022F	5’-CGGGCGGATGCACTATTTT-3’
vcrA1093R	5’-GAATAGTCCGTGCCCTTCCTC-3’

Supplementary Table S3 The averaged coverages for the reductive genes

The averaged coverages of SOLiD 3 tag sequences for the reductive genes of four *Dehalococcoides* spp. (*D. maccartyi* 195, *D. maccartyi* BAV1, *D. maccartyi* CBDB1, and *D. maccartyi* VS) were calculated.

***D. mccartyi* 195**

Gene	Strand	Start	Stop	Coverage
**DET0079 (tceA)**	**-**	**77229**	**78893**	**2042**
**DET0173**	**+**	**167859**	**169391**	**836**
**DET0180**	**+**	**173382**	**174749**	**3718**
**DET0235**	**+**	**226290**	**227762**	**54**
**DET0302**	**+**	**290049**	**291593**	**1**
**DET0306**	**+**	**294215**	**295732**	**0**
**DET0311**	**+**	**298706**	**300253**	**0**
**DET0318**	**+**	**304666**	**306153**	**0**
**DET0876**	**-**	**803653**	**805185**	**0**
**DET1171**	**-**	**1067812**	**1069410**	**0**
**DET1519**	**-**	**1371471**	**1372988**	**0**
**DET1522**	**-**	**1374234**	**1375757**	**905**
**DET1528**	**-**	**1379479**	**1380888**	**3373**
**DET1535**	**-**	**1384117**	**1385601**	**2500**
**DET1538**	**-**	**1386684**	**1388162**	**8**
**DET1545**	**-**	**1392249**	**1393751**	**409**
**DET1559**	**-**	**1404340**	**1405788**	**0**

***D. mccartyi* BAV1**

Gene	Strand	Start	Stop	Coverage
**DehaBAV1_0104**	**-**	**103582**	**105129**	**0**
**DehaBAV1_0112**	**-**	**111779**	**113299**	**0**
**DehaBAV1_0119**	**-**	**117096**	**118538**	**0**
**DehaBAV1_0121**	**-**	**119160**	**120704**	**0**
**DehaBAV1_0173**	**-**	**173374**	**174741**	**279**
**DehaBAV1_0276**	**+**	**288132**	**289703**	**0**
**DehaBAV1_0281**	**-**	**293045**	**294457**	**0**
**DehaBAV1_0284**	**-**	**295198**	**296688**	**0**
**DehaBAV1_0296**	**+**	**305957**	**307495**	**0**
**DehaBAV1_0847 (*bvcA*)**	**-**	**834960**	**836510**	**2432**

D. mccartyi VS

Gene	Strand	Start	Stop	Coverage
DhcVS_82	-	73132	74556	0
DhcVS_88	-	81035	82573	0
DhcVS_96	+	90484	91974	0
DhcVS_99"	+	90484	91974	0
DhcVS_104	-	97423	98994	0
DhcVS_169	+	167053	168420	3162
DhcVS_1260	-	1158576	1160105	0
DhcVS_1263	-	1160729	1162222	0
DhcVS_1291(vcrA)	-	1187299	1188858	3271
DhcVS_1314	-	1211229	1212674	0
DhcVS_1316	-	1213448	1214932	21
DhcVS_1320	-	1217638	1219167	0
DhcVS_1324	-	1221945	1223426	0
DhcVS_1327	-	1224558	1226057	0
DhcVS_1329	-	1226677	1228098	0
DhcVS_1336	-	1235559	1237049	0
DhcVS_1340	-	1240073	1241515	0
DhcVS_1342	-	1241951	1243372	0
DhcVS_1344	-	1243835	1245301	0
DhcVS_1347	+	1246808	1248097	0
DhcVS_1349	-	1248692	1250077	0
DhcVS_1353	-	1251497	1253041	0
DhcVS_1360	-	1257634	1259232	0
DhcVS_1364	-	1261258	1262814	0
DhcVS_1371	-	1267664	1269181	0
DhcVS_1375	-	1272030	1273547	0
DhcVS_1378	-	1274677	1276203	171
DhcVS_1383	-	1279450	1280967	0
DhcVS_1387	-	1282905	1284380	0
DhcVS_1393	-	1287013	1288500	0
DhcVS_1399	-	1295467	1296951	131
DhcVS_1402	-	1298295	1299794	0
DhcVS_1421	-	1322374	1323801	16
DhcVS_1427	-	1329484	1330974	0
DhcVS_1430	-	1332053	1333531	4
DhcVS_1436	-	1337642	1339213	29

D. mccartyi CBDB1

Gene	Strand	Start	Stop	Coverage
cbdb_A80	+	61449	62939	0
cbdb_A84	+	67523	68989	0
cbdb_A88	-	72513	74060	0
cbdb_A96	-	80710	82230	0
cbdb_A187	+	170338	171705	225
cbdb_A238	+	208160	209704	0
cbdb_A243	+	213820	215295	7
cbdb_A1092	+	885869	887467	0
cbdb_A1453	-	1146028	1147515	0
cbdb_A1455	-	1148252	1149745	0
cbdb_A1491	-	1177174	1178622	0
cbdb_A1495	-	1183152	1184669	0
cbdb_A1503	-	1189522	1191102	0
cbdb_A1508	-	1193343	1194734	0
cbdb_A1535	+	1222599	1224086	0
cbdb_A1539	-	1226459	1227841	0
cbdb_A1542	-	1228967	1230430	0
cbdb_A1546	-	1233683	1235200	0
cbdb_A1550	-	1237886	1239412	47
cbdb_A1560	-	1245976	1247517	0
cbdb_A1563	-	1248605	1250002	0
cbdb_A1570	-	1257652	1259175	0
cbdb_A1575	-	1263534	1265051	0
cbdb_A1578	-	1266472	1267989	0
cbdb_A1582	+	1269916	1271391	0
cbdb_A1588	-	1274257	1275744	0
cbdb_A1595	-	1283001	1284485	74
cbdb_A1598	-	1285951	1287450	0
cbdb_A1618	-	1309684	1311111	2
cbdb_A1624	-	1316903	1318390	0
cbdb_A1627	-	1319491	1320969	4
cbdb_A1638	-	1325086	1326588	47
